# How many brain metastases can be treated with stereotactic radiosurgery before the radiation dose delivered to normal brain tissue rivals that associated with standard whole brain radiotherapy?

**DOI:** 10.1002/acm2.13856

**Published:** 2023-01-11

**Authors:** Stewart J. Becker, Evan J. Lipson, Gabor Jozsef, Jason K. Molitoris, Joshua S. Silverman, Joseph Presser, Douglas Kondziolka

**Affiliations:** ^1^ Department of Radiation Oncology University of Maryland School of Medicine Baltimore Maryland USA; ^2^ Bloomberg∼Kimmel Institute for Cancer Immunotherapy and Sidney Kimmel Comprehensive Cancer Center Johns Hopkins University Baltimore Maryland USA; ^3^ Department of Radiation Oncology Weill Cornell Medicine New York New York USA; ^4^ Department of Radiation Oncology New York University Langone Medical Center New York New York USA; ^5^ Department of Radiation Oncology Mount Sinai South Nassau Oceanside New York USA; ^6^ Department of Neurosurgery New York University Langone Medical Center New York New York USA

**Keywords:** brain metastases, gamma knife, radiation toxicity, stereotactic radiosurgery, whole brain radiotherapy

## Abstract

**Introduction:**

Clinical trial data comparing outcomes after administration of stereotactic radiosurgery (SRS) or whole‐brain radiotherapy (WBRT) to patients with brain metastases (BM) suggest that SRS better preserves cognitive function and quality of life without negatively impacting overall survival. Here, we estimate the maximum number of BM that can be treated using single and multi‐session SRS while limiting the dose of radiation delivered to normal brain tissue to that associated with WBRT.

**Methods:**

Multiple‐tumor SRS was simulated using a Monte Carlo – type approach and a pre‐calculated dose kernel method. Tumors with diameters ≤36 mm were randomly placed throughout the contoured brain parenchyma until the brain mean dose reached 3 Gy, equivalent to the radiation dose delivered during a single fraction of a standard course of WBRT (a total dose of 30 Gy in 10 daily fractions of 3 Gy). Distribution of tumor sizes, dose coverage, selectivity, normalization, and maximum dose data used in the simulations were based on institutional clinical metastases data.

**Results:**

The mean number of tumors treated, mean volume of healthy brain tissue receiving > 12 Gy (V12) per tumor, and total tumor volume treated using mixed tumor size distributions were 12.7 ± 4.2, 2.2 cc, and 12.9 cc, respectively. Thus, we estimate that treating 12–13 tumors per day over 10 days would deliver the dose of radiation to healthy brain tissue typically associated with a standard course of WBRT.

**Conclusion:**

Although in clinical practice, treatment with SRS is often limited to patients with ≤15 BM, our findings suggest that many more lesions could be targeted while still minimizing the negative impacts on quality of life and neurocognition often associated with WBRT. Results from this *in silico* analysis require clinical validation.

## 1 INTRODUCTION

Brain metastases (BM) occur in 10%–30% of all cancer patients.^1^ Standard‐of‐care treatment options include stereotactic radiosurgery (SRS) and whole‐brain radiotherapy (WBRT). These techniques can be utilized independently or in combination, administered to intact metastases or to the surgical resection bed in the adjuvant setting. Recent data favor utilization of SRS without WBRT in order to avoid the cognitive deficits associated with WBRT.^2^, ^3^ Administration of WBRT is often considered in cases of disease relapse after SRS.^4^, ^5^, ^6^, ^7^, ^8^, ^9^

Because toxicity associated with WBRT appears to be dose‐dependent, administration of SRS to a sufficient number of BM could decrease selectivity and conformality to such a degree that the dose of radiation delivered to normal brain tissue approaches that associated with WBRT, thereby negating any safety benefit.^10^, ^11^, ^12^, ^13^, ^14^, ^15^, ^16^

In this study, we estimate the maximum number of BM that can be treated using single and multi‐session SRS while limiting the dose of radiation delivered to normal brain tissue to that associated with WBRT. In addition, we estimate the mean dose of radiation delivered to the brain after administration of SRS to single metastases of various sizes.

## 2 MATERIALS AND METHODS

### 2.1 Overview of simulation

We utilized a representative MR image series of an adult brain with normal anatomy.^17^ Simulated tumors were randomly but evenly distributed throughout the brain. Tumor sizes were guided by historical data from a single institution's SRS Treatment Database. Radiation doses were assigned to each tumor using dose distributions created from pre‐calculated dose kernels. The specificity and prescription dose levels of dose distributions were also based on the size of the tumors’ corresponding dosing coverage in the SRS Treatment Database. New tumors and associated dose clouds were placed until the mean dose delivered to normal brain tissues reached 3 Gy (the dose delivered by a standard single fraction of WBRT). Of note, tumor volumes were not included in calculations of normal brain tissue volumes.

### 2.2 Dose kernels and dose clouds

Using the Leksell Gamma Knife Perfexion (Elekta, Stockholm, Sweden) as our SRS device, three‐dimensional (3D) dose distributions were calculated using GammaPlan treatment planning software (10.1) (Elekta, Stockholm, Sweden). A single‐shot dose distribution was created using each of the three GammaKnife Perfexion collimator sizes (4, 8, and 16 mm) at the center of mass of the brain. In addition, a fourth and a fifth dose distribution (24 and 36 mm) were created from a composite of 8‐ and 16‐mm shots. The five resulting dose distributions were exported with 1‐mm resolution in DICOM format into our in‐house simulation program, written in Matlab (MathWorks, Natick, MA). These 3D dose distributions were then used as pre‐calculated dose kernels in the Matlab simulation. Instead of recalculating the doses for every shot placement, the dose kernels were translated to the actual shot positions, summed together, and then renormalized to give each tumor its prescribed dose. From the dose‐volume histogram (DVH) of the summed distribution, the mean dose to the brain was calculated.

### 2.3 Kernel preparation

Before placing a dose kernel, it was normalized based on tumor size (which dictates prescription dose and percentage, maximum dose, selectivity [ratio of the volume of tumor to the volume of tissue receiving the prescription dose]), of the most recent 250 metastases listed in our institution's SRS Treatment Database. (Table 1). Tumors were grouped by size into the following bins: 0–2, >2–4, >4–6, >6–8, >8–12, >12–16, >16–20, >20–24, and > 24–36 mm. For each bin, the average tumor volume and tumor selectivity were calculated. These averages were then used to calculate the average volume receiving the prescription dose for each tumor size bin. We selected a dose kernel that best matched the volumes being treated for each tumor size bin. The normalization isodose line that contained a volume equal to the volume receiving the prescription dose was selected. The average prescription dose from the database was then assigned to that normalization line, which, in turn, determined the maximum dose of the distribution (Table 1). For example, for all tumors in the institutional database >2 mm and ≤4 mm, the average tumor diameter was 3.0 mm, the average selectivity was 0.15, and the average volume of tissue receiving the prescribed dose was 0.7 cm^3^. For the 4‐mm dose kernel, the isodose line that encompassed 0.7 cm^3^ is the 50.6% line. 17.1 Gy was the average prescription dose applied to that line with an average max dose of 33.8 Gy. This average maximum dose is assigned to the d_max_ of that kernel for each tumor placed that was >2 mm and ≤4 mm. In order to calculate the dose over the whole volume of the brain when a target was far from the center of the brain, based on measurements taken in our Gamma Knife Perfexion unit with all the sources in the blocked position, we extended a leakage value of 0.19% to the surrounding tissue. Of note, to account for the lower selectivity associated with tumors that are not spherical and require more than a single SRS shot, we incorporated average selectivity values into the simulation so that our estimate would apply to tumors of various shapes and configurations.

**TABLE 1:** Tumor dose normalization from 250 consecutive metastatic tumors at an institution binned by tumor size

Tumor size	Avg tumor size (mm)	Selectivity	Avg Rx isodose diam (mm)	Avg Rx isodose vol(cm3)	Normalization of kernel that covers vol	Avg Rx dose (Gy)	Max dose (Gy)
0–2 mm	1.8	0.06	4.7	0.4	64.4%	16.3	25.3
>2–4 mm	3.0	0.15	5.6	0.7	50.6%	17.1	33.8
>4–6 mm	4.9	0.25	7.8	2.0	78.3%	17.1	21.8
>6–8 mm	7.0	0.45	9.1	3.2	63.8%	16.6	26.0
>8–12 mm	9.7	0.57	11.7	6.7	84.3%	17.2	20.4
>12–16 mm	13.6	0.58	16.4	18.5	62.5%	17.1	27.4
>16–20 mm	18.0	0.67	20.5	36.1	71.0%	17.0	23.9
>20–24 mm	22.1	0.69	24.9	64.7	57.7%	13.0	22.5
>24–36 mm	28.6	0.75	31.5	130.9	57.8%	14.5	25.1

### 2.4 Selection of tumor size and placement

From all the voxels in the brain contour, a list was created of randomly selected voxels, each designated as a tumor center. Simulated tumors were randomly assigned sizes based on tumor size distributions of the most recent 250 BM listed in our institution's SRS Treatment Database. (Table 2) Simulated tumors were evaluated for overlap. If the distance between the centers of any two tumors was less than the sum of their radii, one of those tumors was removed. However, we allowed overlap of high‐isodose lines from adjacent tumors. Additional simulations were performed utilizing simulated tumors restricted to single size bins (i.e., 0–2, >2–4, >4–6, >6–8, >8–12, >12–16, >16–20, >20–24, and >24–36 mm).

**TABLE 2:** Size and frequency of occurrence of 250 consecutively treated brain metastases (BM) in a single institution's SRS treatment database

Tumor size (mm)	Avg tumor size (mm)	Frequency (%, *N* = 250)
0–2	1.8	3.2
>2–4	3.0	44.6
>4–6	4.9	16.4
>6–8	7.0	10.5
>8–12	9.7	15.9
>12–16	13.6	3.6
>16–20	18.0	2.7
>20–24	22.1	1.4
>24–36	28.6	1.8

### 2.5 Dose summation

We summed dose contributions from treatment of each successive lesion until a mean dose to normal brain tissue of 3 Gy was reached. After simulating treatment to each tumor, we renormalized the dose distributions to account for the radiation that would be delivered to other tumors. Each dose distribution was weighted such that the voxel at the center of each tumor would receive the intended maximum dose. The weights were obtained by solving the following linear equation system:

$$D_{i}=Sd_{ij}*W_{j},i,j=1\text{…}N_{s},i\neq j,$$

where *D_i_* is the dose at the center of *i*th tumor, *d_ij_* is the contribution of the *j*th shot to the *i*th tumor, and *N_s_* is the number of shots so far. These weighted shots were then summed and the process repeated until the calculated mean dose delivered to normal brain tissue was 3 Gy. To mitigate the random nature of the tumor placements, each simulation was run 100 times.

### 2.6 Validation test

In order to validate the above‐described pre‐calculation kernel‐based method, we simulated treatment of 50 tumors using GammaPlan. Tumor locations and sizes were exported to Matlab for recalculation of 3D dose distribution. Resultant data were analyzed in three ways. First, dose profiles generated by GammaPlan and by simulation were compared qualitatively. Second, a γ‐analysis was performed using 3 mm/3% criteria. Third, mean doses delivered based on each plan were compared.

## 3 RESULTS

### 3.1 Simulation results: Clinical tumor size distribution

The average mean and median brain doses were 3.0 Gy ± 0.2 Gy and 2.2 Gy ± 0.2 Gy, respectively. As seen in Table 3, the average number of tumors treated, mean V12 per tumor, and average total tumor volume treated, were 12.7 ± 4.2, 2.2 cc, and 12.9 cc, respectively.

**TABLE 3:** Results of 100 runs to 3 Gy mean whole‐brain dose for scenarios using all tumor sizes and individual tumor sizes ranges

Tumor size (mm)	Mean brain dose (Gy)	Median brain dose (Gy)	Avg # of tumors	Avg V12/tumor (cc)	Tumor vol treated (cc)	Whole‐brain dose/tumor (Gy)
0–36	3.0(.2)	2.2(.2)	12.7(4.2)	2.2	12.9	0.2
0‐2	3.0(.2)	2.7 (0.04)	76.2 (1.7)	0.1	0.1	0.04 (0.009)
>2–4	3.0(.2)	2.7 (0.04)	55.3 (1.6)	0.2	1.2	0.05 (0.0015)
>4‐6	3.0(.2)	2.6 (0.06)	29.8 (1.2)	0.5	3.2	0.1 (0.004)
>6–8	3.0(.2)	2.6 (0.07)	24.4 (1.4)	0.6	5.5	0.12 (0.007)
>8‐12	3.0(.2)	2.3 (0.1)	9.38 (0.8)	2.9	7.8	0.3 (0.02)
>12–16	3.0(.2)	2.3 (0.2)	6.77 (0.6)	3.6	20.4	0.4 (0.04)
>16‐20	3.0(.2)	2.2 (0.2)	4.77 (0.5)	7.5	29.7	0.6 ((0.06)
>20–24	3.0(.2)	2.3 (0.2)	4.87 (0.8)	5.8	66.9	0.6 (0.1)
>24–36	3.0(.2)	2.0 (0.4)	2.68 (0.6)	13.5	97.6	1.2 (0.3)

### 3.2 Simulation results: Single tumor sizes

Runs of single tumor sizes were calculated to determine the average whole‐brain dose delivered per tumor. For tumor sizes of 0–2, > 2‐4, > 4–6, > 6‐8, > 8–12, > 12‐16, > 16–20, > 20‐24, and > 24–36 mm, whole‐brain doses per tumor were 0.04, 0.05, 0.1, 0.12, 0.3, 0.4, 0.6, 0.6, and 1.2 Gy, respectively. Table 3 displays these results along with the mean and median dose to the brain and the V12 per tumor treated.

### 3.3 Validation test

**FIGURE 1:**
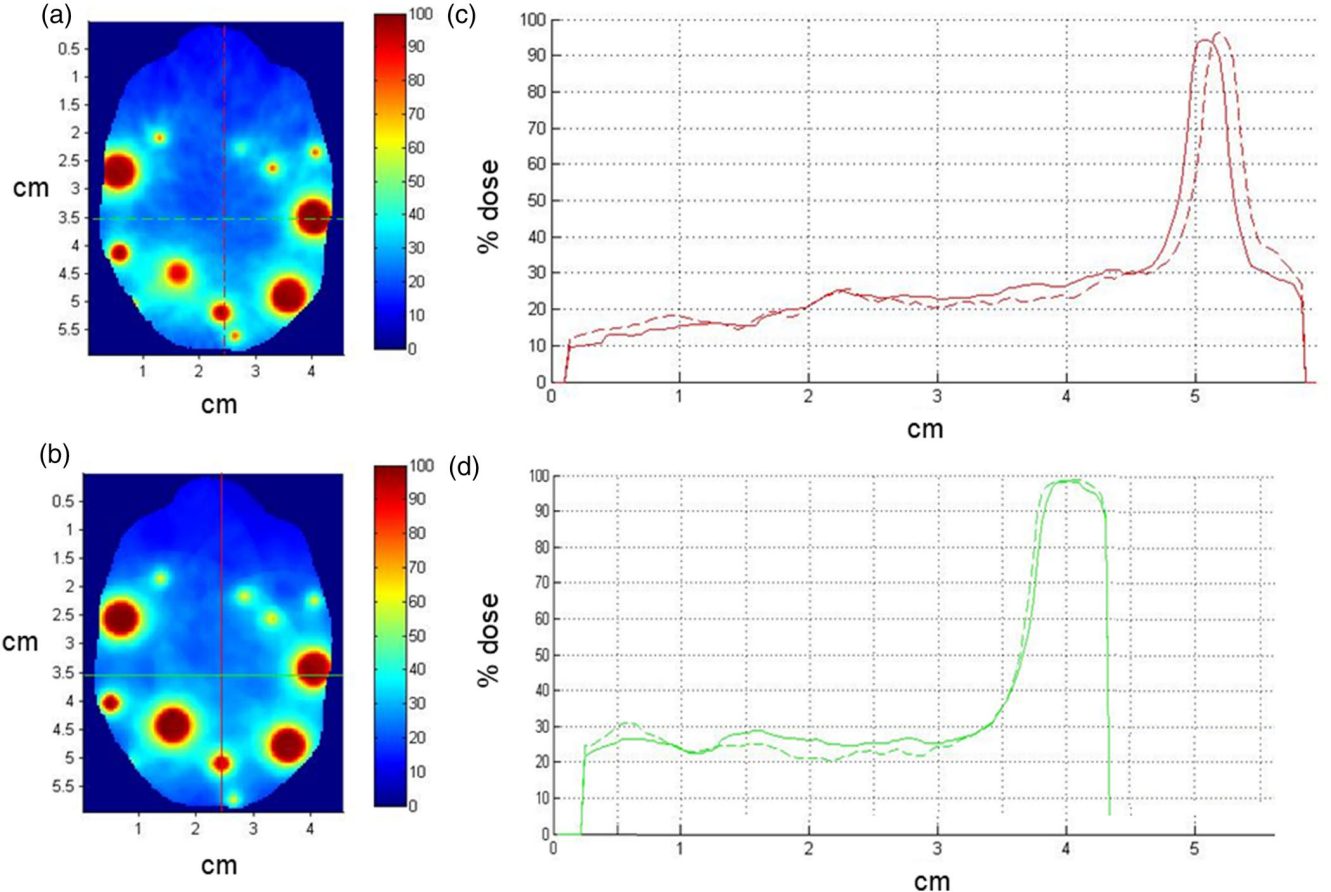
Dose profiles validating dose calculation accuracy of the simulation method used in the current study. Panels (a) and (b) illustrate planar isodose distributions from the GammaPlan and the simulation, respectively. Panels (c) and (d) illustrate equivalency of the vertical and horizontal dose profiles from GammaPlan (dashed line) and simulation (solid line)

Qualitative depictions of dose profiles generated by GammaPlan and by simulation appeared to be equivalent (Figure 1). γ‐analysis revealed a 98.2% pass rate using 3 mm/3% criteria. Mean doses delivered based on each plan were equivalent (4.76 Gy).

## 4 DISCUSSION

In recent years, SRS has been administered to patients to treat an increasing number of BM and minimize the negative impacts on quality of life and neurocognition often associated with WBRT. However, guidelines regarding the number of metastases that can be safely treated are lacking, and practice patterns vary widely across the globe.^18^ One recent analysis suggests acceptable safety and efficacy profiles associated with administration of SRS to ≤15 metastases.^19^ Another retrospective study estimated the radiation dose delivered to normal brain as a function of the total tumor volume treated with SRS.^20^ To complement these emerging data, we used a novel approach to estimate that treating 12–13 tumors per day over 10 days would deliver the dose of radiation to healthy brain tissue typically associated with a standard course of WBRT (3 Gy x 10 fractions). This discovery applies to patients undergoing first‐line therapy as well as to patients with new tumors that appear after an original SRS course, a scenario in which administration of WBRT is frequently entertained.^21^

The dose of radiation delivered to normal brain tissue during SRS increases as the volume of the treated tumor increases (Table 3). An estimate of the radiation dose delivered to normal brain can be calculated by multiplying the number of tumors of a given size by the dose per tumor value given in Table 3 and summing the total.

One limitation of our Gamma Knife‐based analysis is that our estimates may not be extrapolatable to other radiotherapy modalities such as linac‐based techniques (e.g., cones, volumetric‐modulated arc therapy [VMAT], tomotherapy, robotic methods, single isocenter multiple targets [SIMT]). Future studies might involve similar analyses based on alternative treatment approaches. Another study limitation is the creation of a simulation model from a single institution's SRS Treatment Database. Although the model includes 250 lesions, the dataset may not be representative of tumor locations and doses administered at other institutions. Finally, while the analyses performed in our study do not explicitly account for hippocampal avoidance techniques, the prescription dose to the treated brain is the same for both hippocampal‐sparing and standard WBRT.^22^ Indeed, the goal of the study is to model the number of BM that can be treated before approaching the radiation dose delivered to normal brain regardless of whether or not the hippocampus is included.

In sum, our results suggest that treating 12–13 tumors per day over 10 days would deliver the dose of radiation to healthy brain tissue typically associated with a standard course of WBRT. Clinical exploration of our findings is required in order to address logistical concerns (e.g., time required to treat several dozen lesions with SRS) and potential risks of avoiding WBRT (often administered to treat tumors that are not yet visible on imaging) in favor of short‐term follow‐up imaging and repeat SRS. As techniques designed to minimize the negative impacts on quality of life and neurocognition often associated with WBRT evolve, the differences in morbidity between these new approaches and SRS may narrow.

## AUTHOR CONTRIBUTIONS

All authors contributed to either drafting or critical editing of the manuscript's intellectual content well as final approval of the documents. Stewart Becker, Gabor Josef, Evan Lipson, and Doug Kondziolka made substantial contribution to design of the work. Stewart Becker, Joseph Presser, and Gabor Jozsef made substantial contribution to data acquisition and analysis. Stewart Becker, Evan Lipson, Jason Molitoris, Joshua Silverman, and Douglas Kondziolka made substantial contribution to interpretation of data for the work.

## CONFLICT OF INTEREST

No conflicits of interest

## References

[1] DeVita VT , Hellman S , Rosenberg SA . Cancer, Principles & Practice of Oncology. 7th ed. Lippincott Williams & Wilkins; 2005.

[2] Chang EL , Wefel JS , Hess KR , et al. Neurocognition in patients with brain metastases treated with radiosurgery or radiosurgery plus whole‐brain irradiation: a randomised controlled trial. Lancet Oncol. 2009;10(11):1037‐1044. doi:10.1016/S1470-2045(09)70263-3 19801201

[3] Gemici C , Yaprak G . Whole‐brain radiation therapy for brain metastases: detrimental or beneficial? Radiat Oncol. 2015;10:153. doi:10.1186/s13014-015-0466-9 26215106 PMC4517629

[4] Aoyama H , Shirato H , Tago M , et al. Stereotactic radiosurgery plus whole‐brain radiation therapy vs. stereotactic radiosurgery alone for treatment of brain metastases: a randomized controlled trial. JAMA. 2006;295(21):2483‐2491. doi: 295/21/2483 16757720 10.1001/jama.295.21.2483

[5] Scoccianti S , Ricardi U . Treatment of brain metastases: review of phase III randomized controlled trials. Radiother Oncol. 2012;102(2):168‐179. doi:10.1016/j.radonc.2011.08.041 21996522

[6] Hatiboglu MA , Tuzgen S , Akdur K , Chang EL . Treatment of high numbers of brain metastases with Gamma Knife radiosurgery: a review. Acta Neurochir (Wien). 2016;158(4):625‐634. doi:10.1007/s00701-016-2707-6 26811300

[7] Gemici C , Yaprak G , et al. SRS with or without whole‐brain radiation therapy for those with 1 to 4 brain metastases: in regard to Sahgal et al. Int J Radiat Oncol Biol Phys. 2015;92(4):947‐948. doi:10.1016/j.ijrobp.2015.04.012 26104947

[8] Vellayappan B , Sahgal A , Chang EL , Lo SS . Commentary: clinical outcomes of upfront stereotactic radiosurgery alone for patient with 5 to 15 brain metastases. Neurosurgery. 2019;85(2):E247‐E48. doi:10.1093/neuros/nyy278 29982791

[9] Sahgal A , Ruschin M , Ma L , Verbakel W , Larson D , Brown PD . Stereotactic radiosurgery alone for multiple brain metastases? A review of clinical and technical issues. Neuro Oncol. 2017;19(Suppl_2):ii2‐ii15. doi:10.1093/neuonc/nox001 28380635 PMC5463499

[10] Rush S , Elliott RE , Morsi A , et al. Incidence, timing, and treatment of new brain metastases after Gamma Knife surgery for limited brain disease: the case for reducing the use of whole‐brain radiation therapy. J Neurosurgery. 2011;115(1):37‐48. doi:10.3171/2011.2.JNS101724 21417707

[11] Serizawa T , Hirai T , Nagano O , et al. Gamma knife surgery for 1–10 brain metastases without prophylactic whole‐brain radiation therapy: analysis of cases meeting the Japanese prospective multi‐institute study (JLGK0901) inclusion criteria. J Neuro‐Oncology. 2010;98(2):163‐167. doi:10.1007/s11060-010-0169-x 20411300

[12] Yamamoto M , Ide M , Nishio S , Urakawa Y . Gamma Knife radiosurgery for numerous brain metastases: is this a safe treatment? Int J Radiat Oncol Biol Phys. 2002;53(5):1279‐1283. doi:S0360301602028559 12128130 10.1016/s0360-3016(02)02855-9

[13] Grandhi R , Kondziolka D , Panczykowski D , et al. Stereotactic radiosurgery using the Leksell Gamma Knife Perfexion unit in the management of patients with 10 or more brain metastases. J Neurosurgery. 2012;117(2):237‐245. doi:10.3171/2012.4.JNS11870 22631694

[14] Yamamoto M , Kawabe T , Sato Y , et al. Stereotactic radiosurgery for patients with multiple brain metastases: a case‐matched study comparing treatment results for patients with 2–9 versus 10 or more tumors. J Neurosurgery. 2014;121(Suppl 2):16‐25. doi:10.3171/2014.8.GKS141421 25434933

[15] Kowalski ES , Remick JS , Sun K , et al. Immune checkpoint inhibition in patients treated with stereotactic radiation for brain metastases. Radiat Oncol. 2020;15(1):245. doi:10.1186/s13014-020-01644-x 33109224 PMC7590444

[16] Remick JS , Kowalski E , Khairnar R , et al. A multi‐center analysis of single‐fraction versus hypofractionated stereotactic radiosurgery for the treatment of brain metastasis. Radiat Oncol. 2020;15(1):128. doi:10.1186/s13014-020-01522-6 32466775 PMC7257186

[17] Cosgrove KP , Mazure CM , Staley JK . Evolving knowledge of sex differences in brain structure, function, and chemistry. Biol Psychiatry. 2007;62(8):847‐855. doi:10.1016/j.biopsych.2007.03.001 17544382 PMC2711771

[18] Sandler KA , Shaverdian N , Cook RR , et al. Treatment trends for patients with brain metastases: does practice reflect the data? Cancer. 2017;123(12):2274‐2282. doi:10.1002/cncr.30607 28178376

[19] Hughes RT , Masters AH , McTyre ER , et al. Initial SRS for patients with 5 to 15 brain metastases: results of a multi‐institutional experience. Int J Radiat Oncol Biol Phys. 2019;104(5):1091‐1098. doi:10.1016/j.ijrobp.2019.03.052 30959122

[20] Rivers C , Tranquilli M , Prasad S , et al. Impact of the number of metastatic tumors treated by stereotactic radiosurgery on the dose to normal brain: implications for brain protection. Stereotact Funct Neurosurg. 2017;95(5):352‐358. doi:10.1159/000480666 29017157

[21] Maranzano E , Trippa F , Casale M , et al. Reirradiation of brain metastases with radiosurgery. Radiother Oncol. 2012;102(2):192‐197. doi:10.1016/j.radonc.2011.07.018 21880387

[22] Brown PD , Gondi V , Pugh S , et al. Hippocampal avoidance during whole‐brain radiotherapy plus memantine for patients with brain metastases: phase III trial NRG oncology CC001. J Clin Oncol. 2020;38(10):1019‐1029. doi:10.1200/JCO.19.02767 32058845 PMC7106984

